# The role of plant growth promoting rhizobacteria in strengthening plant resistance to fluoride toxicity: a review

**DOI:** 10.3389/fmicb.2023.1271034

**Published:** 2023-10-10

**Authors:** Anamika Singh, Virendra Kumar Yadav, Hemant Gautam, Lokendra Rathod, Rajendra Singh Chundawat, Gulab Singh, Rakesh Kumar Verma, Dipak Kumar Sahoo, Ashish Patel

**Affiliations:** ^1^School of Liberal Arts and Sciences, Mody University of Science and Technology, Sikar, Rajasthan, India; ^2^Department of Life Sciences, Hemchandracharya North Gujarat University, Patan, Gujarat, India; ^3^CSIR-Institute of Genomics and Integrative Biology, New Delhi, India; ^4^ICMR-National Institute for Research in Environmental Health, Bhopal, Madhya Pradesh, India; ^5^Department of Veterinary Clinical Sciences, College of Veterinary Medicine, Iowa State University, Ames, IA, United States

**Keywords:** rhizosphere, fluoride transporters, PGPR, defense systems, bioremediation, chemical fertilizers

## Abstract

A wide variety of bacteria are present in soil but in rhizospheric area, the majority of microbes helps plant in defending diseases and facilitate nutrient uptake. These microorganisms are supported by plants and they are known as plant growth-promoting rhizobacteria (PGPR). The PGPRs have the potential to replace chemical fertilizers in a way that is more advantageous for the environment. Fluoride (F) is one of the highly escalating, naturally present contaminants that can be hazardous for PGPRs because of its antibacterial capacity. The interactions of F with different bacterial species in groundwater systems are still not well understood. However, the interaction of PGPR with plants in the rhizosphere region reduces the detrimental effects of pollutants and increases plants’ ability to endure abiotic stress. Many studies reveal that PGPRs have developed F defense mechanisms, which include efflux pumps, Intracellular sequestration, enzyme modifications, enhanced DNA repair mechanism, detoxification enzymes, ion transporter/antiporters, F riboswitches, and genetic mutations. These resistance characteristics are frequently discovered by isolating PGPRs from high F-contaminated areas or by exposing cells to fluoride in laboratory conditions. Numerous studies have identified F-resistant microorganisms that possess additional F transporters and duplicates of the well-known targets of F. Plants are prone to F accumulation despite the soil’s low F content, which may negatively affect their growth and development. PGPRs can be used as efficient F bioremediators for the soil environment. Environmental biotechnology focuses on creating genetically modified rhizobacteria that can degrade F contaminants over time. The present review focuses on a thorough systemic analysis of contemporary biotechnological techniques, such as gene editing and manipulation methods, for improving plant-microbe interactions for F remediation and suggests the importance of PGPRs in improving soil health and reducing the detrimental effects of F toxicity. The most recent developments in the realm of microbial assistance in the treatment of F-contaminated environments are also highlighted.

## 1. Introduction

Numerous types of bacteria live in the rhizosphere, most of which can protects plants from pathogenic invasions and make it easier for plants to absorb nutrients from the soil (Kour et al., 2019). Various studies have shown that the host plant considerably affects the bacterial community in its rhizosphere. Phylogenetically diverse microorganisms, such as viruses, nematodes, fungi, protists, bacteria, and archaea, can all be found in the rhizosphere (Ling et al., 2022). Rhizospheres of plants generally contain a wide range of soil microorganisms (Yang et al., 2022; Nie et al., 2023). Some of these are helpful to the plants since they encourage growth. These microorganisms are referred to as plant growth-promoting rhizobacteria (PGPR) and have the potential to replace chemical fertilizers (Bhattacharyya et al., 2020).

The majority of the PGPRs belong to genera like *Pantoae, Alcaligenes, Arthrobacter, Pseudomonas, Rhodococcus, Serratia, Azoarcus, Azospirillum, Azotobacter, Herbaspirillum, Stenotrophomonas, Lactobacillus, Paenobacillus, Beijerinckia, Burkholderia, Derxia, and Zoogloea* are some of the bacteria that have been identified (Vega-Celedón et al., 2021). Although tight interactions between soil, plants, and microbes are a complex phenomenon that occurs in natural ecosystems, it is challenging to measure how they affect plant growth, health, and production (Uruén et al., 2020; Nadarajah and Abdul Rahman, 2021).

Interactions of plants with PGPR in the rhizosphere area help to maintain soil fertility and plant health. A number of pollutants in the rhizosphere, have a detrimental effect on soil fertility and plant productivity (Vecchiato et al., 2021). In the rhizospheric region of soil, PGPR interacts with plants and protect them from the harmful effect of various pollutants by inducing their ability of abiotic stress tolerance (Khatoon et al., 2020).

Two studies shows that *Bacillus* sp. are suitable for abiotic environment, *Bacillus subtilis* ER-08 (BST) as a stress-resilient, multifunctional plant growth-promoting rhizobacterial isolate and this strain has also been found to increase the development of fenugreek (*T. foenum-graecum*L.) under salt and drought stress and solubilizing phosphate and producing ACC deaminase, siderophore, and IAA helps *Bacillus* sp. promote tomato growth in both non-stressed and salt-stressed environment. Increased amounts of osmoregulatory proline and soluble sugar, as well as ROS scavenging enzymes, were also connected to tomato seedlings’ ability to withstand salt stress (Patani et al., 2023; Patel et al., 2023a,b).

Several studies demonstrate the effect of the PGPR consortiums more efficient than single rhizobacteria for enhancing abiotic stress tolerance in plants (Singh et al., 2023; Wang et al., 2023; Patel et al., 2023a,b).

Among all the toxins, F is one of the toxic elements that cause detrimental effects to plants and other microorganisms. High F levels are a major threat due to their toxicological and geo-environmental concerns. A potent rhizobacterial species must be quickly developed in order to decrease the dangerous consequences of F accumulation in plants (Aggarwal and Bhushan, 2019; Chatterjee et al., 2020; Han et al., 2021).

Duffin et al. (2022) reported that F inhibits microbial development and organic matter decomposition, especially in quantities far higher than its natural values in soil.

F concentrations and the composition of the bacterial population in shallow groundwater were found to be correlated in the Qiji region of Northern China (Zhang et al., 2019). The amounts of Total Organic Carbon (TOC) and F in groundwater have a substantial impact on the bacterial communities. The result illuminates the biogeochemical processes of F and other elements in groundwater and suggests that F concentration should be considered while examining microbial response in an F-rich environment (Kashif et al., 2023).

Many organisms including PGPR have developed fluoride defense systems (Weerasooriyagedara et al., 2020). F riboswitch is one of the defense systems in PGPRs. In a study, *Bacillus cereus* F riboswitch was used in base-pair opening dynamics research with and without ligands to better understand the molecular mechanism of gene regulation in F riboswitch. The finding suggests that the F riboswitch controls gene expression via a two-step mechanism that includes conformational changes which is caused by Mg^2+^ (Lee H.-J. et al., 2021; Lee J. et al., 2021).

These resistance characteristics are typically discovered by separating organisms from F rich environment or in a laboratory setting, cells were exposed to F. Several studies have revealed F-resistant microorganisms with increased F transporters and copies of well-known targets for F (Faraj et al., 2019; Zhao et al., 2019; McIlwain et al., 2021).

Roshni, 2022 reported that F inhibited the activity of protease, alkaline phosphatase, and dehydrogenase enzymes in microorganisms.

PGPR from the genera *Azotobacter, Flavobacterium, Micrococcus, Bacillus, Pseudomonas, Acinetobacter, Streptomyces,* and *Streptococcus* have been found in the rhizosphere (Cochard et al., 2022). *Streptococcus* sp., *Pseudomonas* sp., *Bacillus* sp., and *Acinetobacter* sp. are a few prokaryotes that may survive in environments with high F compound concentrations. This is because of an old system made up of F-specific riboswitches and frequently linked proteins like CrcB (Li et al., 2018; Speed et al., 2018; Lee H.-J. et al., 2021; Lee J. et al., 2021).

The FEX membrane transport protein functions as the primary F defense mechanism in plants. Both prokaryotes (Fluoride channel; Fluc) and fungi (Fluoride Exporter; FEX) have F-specific ion transporters that effectively export fluoride to the extracellular environment. All around the plant kingdom, FEX homologs have been recognized. FEX is conserved in both yeast and plants (Ma et al., 2019; Tausta et al., 2021).

Zhu et al., 2019 reported an increase in germination rate in several lines when fluoride was used. Their finding indicates the optimal concentration or localization of FEX within the plant.

Although each plant has a different level of F tolerance by which they can escape its threatening progression.

According to Pelc et al. (2020) the Dalewar, Arkadia, and Tobak winter wheat cultivars were subjected to sodium fluoride (NaF) treatment, and the results revealed that NaF decreased germination, root growth, and the activity of antioxidant enzymes. These effects have been made worse at higher concentrations of NaF (Pelc et al., 2020; Li et al., 2021). For the last 6 years researchers have been working on the identification of F-tolerant PGPR and their diverse mechanism of tolerance, so that such PGPR can be used as a natural cure for F toxicity and protect crop health from the damaging effects of F (Liao et al., 2018; Mukherjee and Halder, 2018; Sahoo and Goli 2018; Ong et al., 2020; Pelc et al., 2020; Shanker et al., 2020; Chellaiah et al., 2021; Lee H.-J. et al., 2021; Lee J. et al., 2021; Mothe et al., 2021; Mushtaq et al., 2021).

In the current review, the authors have emphasized the current trends of in-depth systemic analysis of contemporary biotechnological approaches, for improving plant-microbe interactions for F degradation, such as gene manipulation and editing approaches, calling attention to the most recent advancements in the field of microbial-endorsed treatment of F contaminated ecosystems. Furthermore, here authors emphasized that with the help of additional investigation and knowledge of the molecular mechanisms behind F bioremediation, more capable strains will be identified for F-contaminated areas.

## 2. Mechanism of PGPR in plant growth stimulation

**Figure 1:**
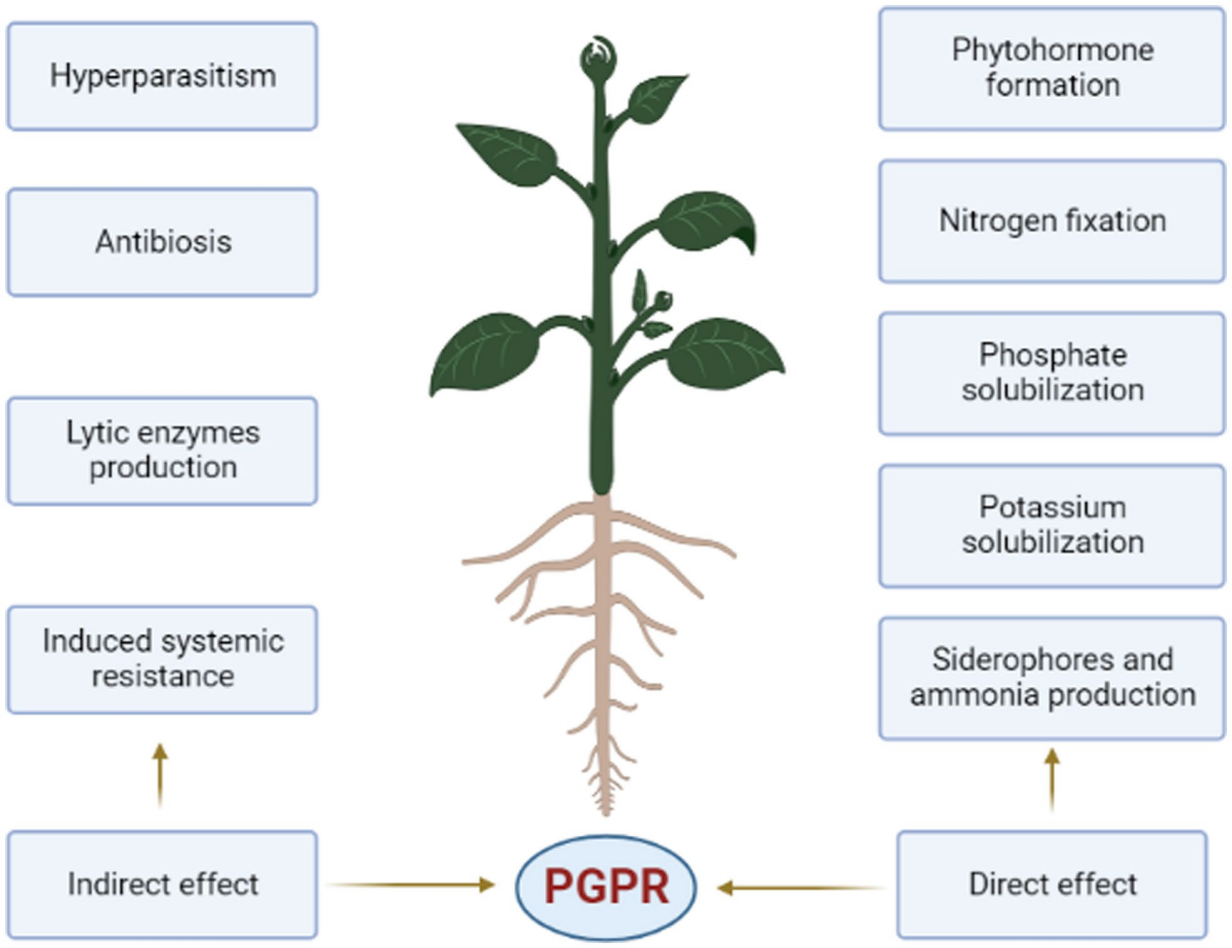
Direct and indirect mechanisms of PGPR’s.

PGPR activity in the rhizosphere is predominantly caused by two different types of processes. Figure 1 depicts the direct and indirect categories. Both methods of PGPR activity are critical to long-term agricultural crop productivity (Patil et al., 2019; Aioub et al., 2022).

Constantia and Ferniah (2020) studied that, by releasing various beneficial compounds like phosphates, silicon, potassium, and zinc, as well as PGPR encourages plant growth by absorbing biologically fixed nitrogen, chelating iron, and other micronutrients, and increasing the amount of geospheric oxygen that is available. This stimulates the formation of aeriform biomass, growth of the roots, and lengthening of the stem. PGPR produces ethylene, auxins, cytokinin, indoleacetic acid, gibberellins, and auxins phytohormone (Del Orozco-Mosqueda et al., 2023).

Tiwari et al. (2018), reported that synthesis of the enzyme 1-aminocyclopropane 1-carboxylate deaminase (ACC), increases root density and length while lowering ethylene levels in crop roots. PGPR indirectly alters the rhizospheric environment, creates systemic resistance, and boosts the plant’s inherent resilience (Adrees et al., 2019).

PGPR released substances like siderophore, pigments, antibiotics, organic acids, water-soluble vitamins, and various volatile organic compounds like monoterpene alcohols and these substances activate the plant’s defense mechanism against various pathogenic microorganisms and promote the synthesis of physical and chemical barriers against abiotic stress (Chandran et al., 2021).

PGPR interferes with the quorum sensing signal and prevents harmful bacteria from developing biofilms around plant roots and in turn plants help PGPR to become more competitive in niche colonization (Hartmann, 2020). Additionally, contaminated soils can be cleaned up with PGPR as investigated (Vaishnav et al., 2022).

Phale (2018) describes the multifunctionality of PGPR and its high demand in agroforestry management. Furthermore, according to Lindemann (2019), PGPRs are significant ecosystem service providers because of their interaction with numerous microbial populations and multifunctional activities. Sylia et al. (2022) explore how the intricately interwoven PGPRs network influences the vegetational biome and soil microfauna by controlling the transmission and circulation of energy and resources across a whole ecosystem (Sylia et al., 2022).

Since all rhizobacteria have the capacity to fix atmospheric nitrogen, they are all referred to as rhizobacteria. Each of the direct and indirect mechanisms may be present or absent in all rhizobacteria (Singh et al., 2023) despite the fact that both the direct and indirect mechanisms (Figure 1) of PGPR benefit plant health (Mahmud et al., 2020).

PGPR is the finest nitrogen source for long-term crop production. *Proteobacteria, Alphaproteobacteria, Rhodospirillales, Acetobacteraceae, Actinobacteria, Micrococcales, Microbacteraceae,* and *Roseomonas* (Zhang et al., 2020), *Burkholderiatropica, Achromobacterinsolitus, and Acetobacterdiazotrophicus*are some nitrogen-fixing microorganisms (Singh et al., 2020).

## 3. Bioaccumulation of fluoride and its mechanism in plants

F contamination of the environment, primarily caused by geological processes but occasionally also resulting from anthropogenic activity, F is absorbed by plants from the contaminated soil and water, which leads to abiotic stress and interference with vital physiological and biochemical processes (Makete et al., 2022).

F in soil-water systems is primarily caused by volcanic eruption, weathering, and rock leaching (Yadav et al., 2021). F has been identified as a mobile component in soil. Additionally, the relative movement showed that soil rather than rocks played a larger role in the release of fluoride into groundwater. Despite being primarily emitted into the atmosphere, water contamination is a significant issue. F contamination in water is due to its excess atmospheric emissions, dumping of contaminated wastewater in water bodies, and chemical weathering are some ways that brought F to the soil surface of plants (Singh et al., 2018; Kabir et al., 2019; Yadav et al., 2019).

### 3.1. Bioaccumulation of fluoride in plants

A common phytotoxic plant pollutant is fluoride. Fluoride is taken by plants from soil and water through their roots and leaves (Gadi et al., 2020). There are numerous influences on fluoride accumulation in plants, including soil fluoride content, and soil and plant species characteristics (Makete et al., 2022). Root biochemistry, morphology, and physiological behavior are all affected by fluoride buildup in the soil. F toxicity in plants depends on the amount, frequency, and length of exposure as well as the genotype of the plant (Sharma and Kaur, 2018). On the other hand, F exposure, even in small doses, has a negative effect on crop species and other plants’ growth and productivity (Sahariya et al., 2022).

F accumulation in plants is a severe concern, impacting their growth and development (Sahariya et al., 2021). F accumulation in plants is affected by a number of factors, including soil F content, plant species, and soil qualities (Banerjee et al., 2020).

The ability of the tea plant to hyper-accumulate F in its leaves suggests that F addition can enhance pectin content and dimethyl esterification, resulting in greater absorption of metal cations and chelation of F in the cell wall via metal ion action (Luo et al., 2021).

**Figure 2:**
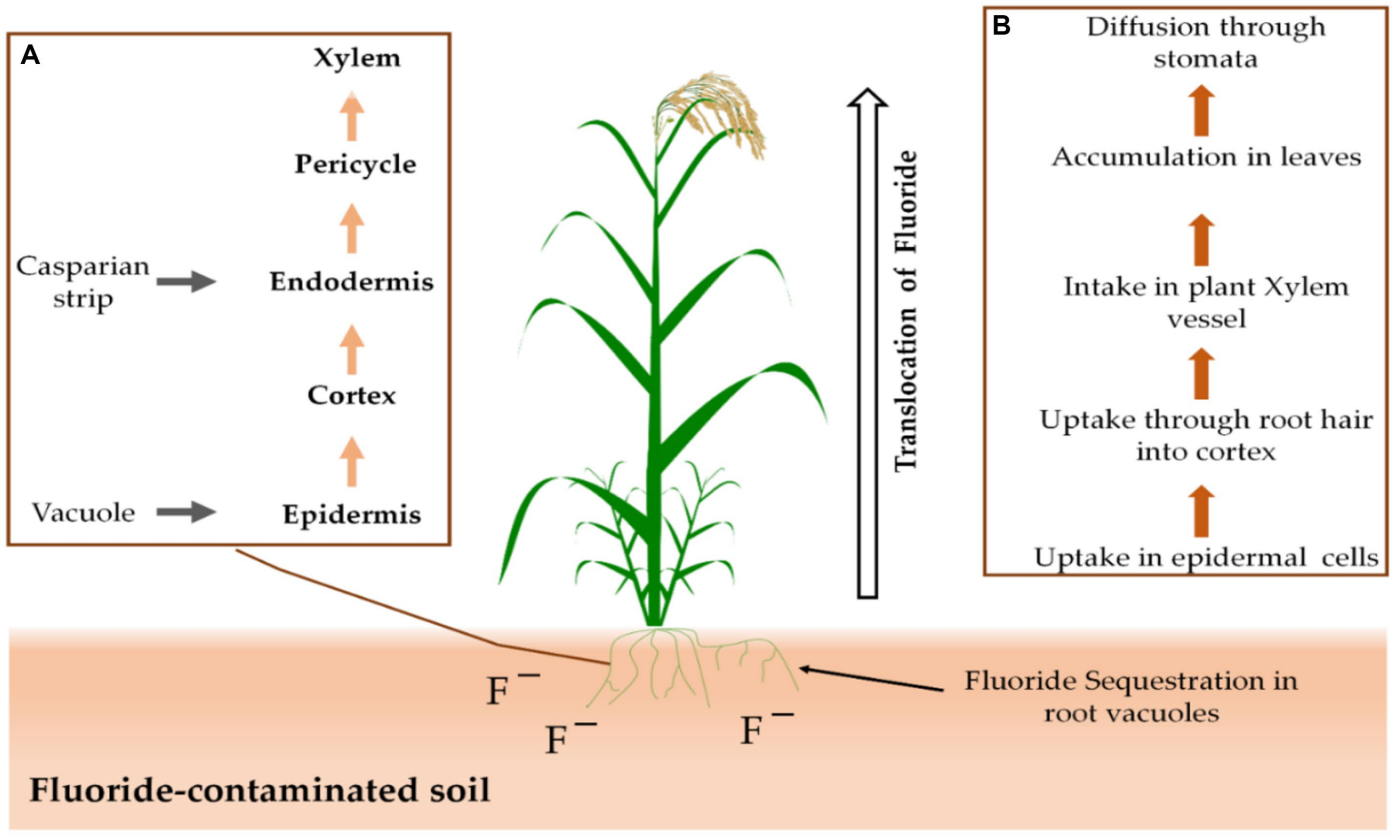
There are two distinct processes at play: The first **(A)** is the uptake mechanism of F that occurs in the roots, while the second **(B)** involves the overall movement of F from the roots up to the shoots.

Stomata or the root system of plants allow them to take up F from the air or soil. Through the secondary roots’ cortex and epidermis, F ions are transported directly into the xylem and phloem (Figure 2; Kumar et al., 2021). Following that, stomata on the plants allow F to diffuse out of the plants (Sharma and Kaur, 2018). Many agricultural plants are inhibited in their growth and metabolism by excess F, while some have an inbuilt capacity for F tolerance (Gadi et al., 2020).

### 3.2. Effect of fluoride on plant health

Environmental F levels, particularly in surface and subsurface irrigation waters, can directly affect the germination of seeds and subsequent plant growth (Almeida Rodrigues et al., 2022). F toxicity leads to a decrease in root protrusion as well as impairment in the mobilization of carbohydrates that reduce healthy growth and embryonic axis development (Chatterjee et al., 2020). As a result, F may build up in vegetative tissues and edible cereal grains, posing a direct hazard to the food chain (Kadiresan and Khanal, 2018).

According to Singh and Roychoudhury (2020), seedlings experienced severe oxidative stress after being exposed to two different F concentrations, 25 and 50 mg L^−1^ NaF, which led to an increase in F accumulation and growth inhibition, as well as decreases in tissue biomass, the size of the roots and shoots, the amount of chlorophyll, the amount of H_2_O_2_ present, and the amount of lipid peroxidation (malondialdehyde content and lipoxygenase activity), and protein carbonylation.

Pelc et al. (2020) studied the effect of NaF on three winter wheat cultivars (Dalewar, Arkadia, and Tobak), and their results showed that NaF reduced germination, root development, and catalase (CAT) activity. The increase in NaF levels has exacerbated these consequences. Sodium fluoride inhibited catalase (CAT) activity significantly at all doses (Pelc et al., 2020).

Prolonged exposure to fluoride at a concentration of 40 mg/L, the photosynthetic pigments and antioxidant enzymes such as glutathione (GSH), ascorbic acid (AsA), and superoxide dismutase (SOD) were sharply decreased in *Hydrilla verticillate* (Gao et al., 2018). Furthermore, when the concentration of F increased, the observed effect became stronger.

According to Sharma and Kaur (2019) at high concentrations of 50 ppm for 24 and 72 h, maximum Glutathione reductase (GR) activity was found. However, for exposure times of 120 and 168 h, activity was decreased at 25 and 20 ppm concentrations of F. When under stress, H_2_O_2_ is scavenged via Ascorbate peroxidase (APOX) activity which is considerably increased at high concentrations of fluoride in *Spirodela polyrhiza* (Sharma and Kaur, 2019).

**Figure 3:**
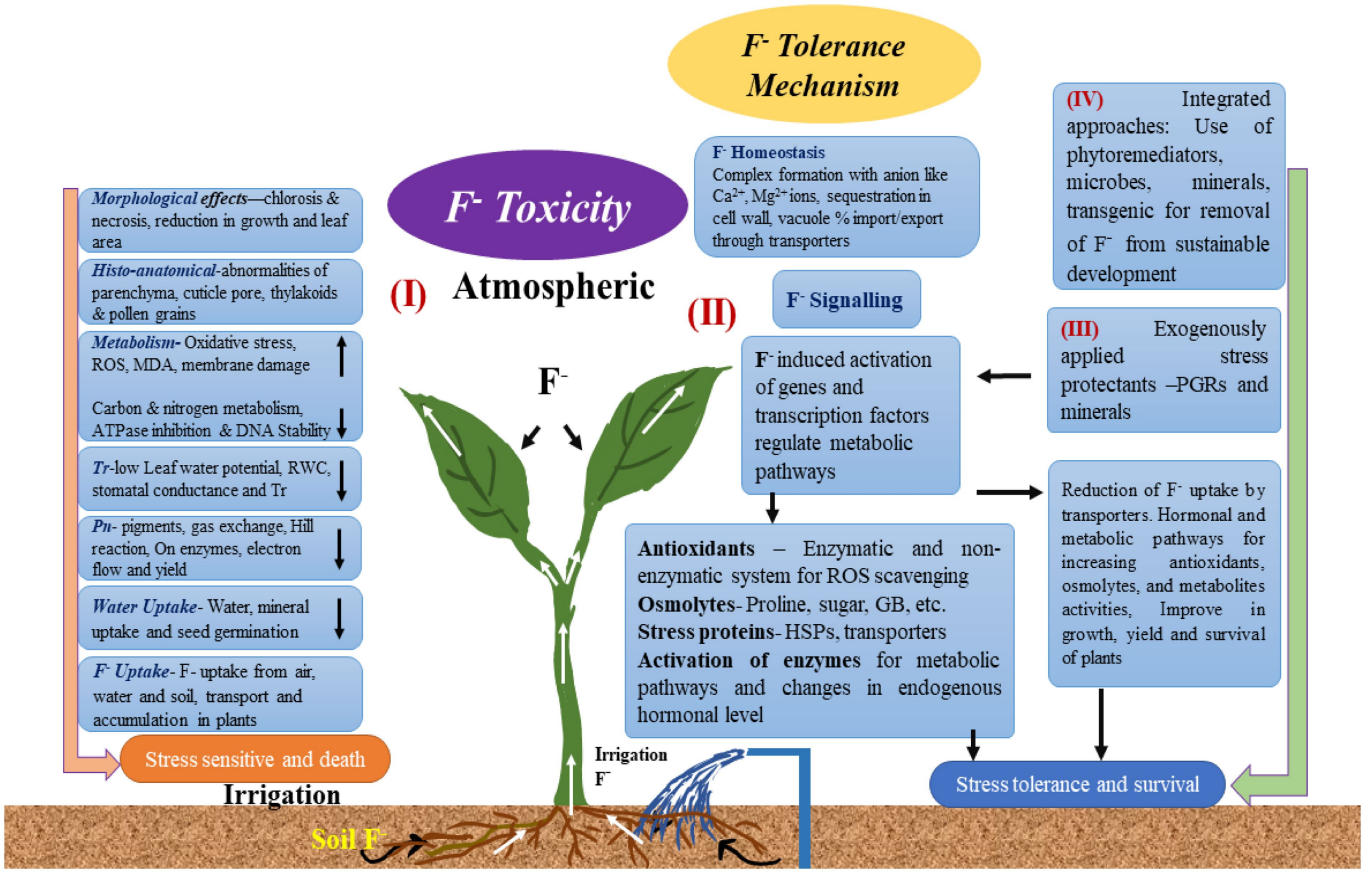
Fluoride bioaccumulation in plants and its negative effects.

Although some plant species are naturally F-tolerant, excessive F impairs the growth and metabolism of many crop species (Figure 3; Gadi et al., 2020).

### 3.3. Fluoride tolerance mechanism of plants

Plants can reduce the stress caused by metabolic disturbances to some extent by activating their defense mechanism. Plants defense system becomes hyperactive to reduce the harmful effects of Reactive Oxygen Species (ROS) (Caverzan et al., 2019; Huang et al., 2019; Hasanuzzaman et al., 2020).

The main F defense mechanism in plants is the action of the FEX (Fluoride Exporter) membrane transport protein. Plant FEX plays a conserved role in F tolerance. A CRISPR/Cas9-generated mutation in *Arabidopsis thaliana*, FEX makes the plant sensitive to low F concentrations (100 μM) at all stages of development. Pollen is particularly vulnerable and unable to develop even at extremely low F levels in the growing media (Tausta et al., 2021). Sequence alignment was used to find FEX homologous genes in nine plants (Banerjee and Roychoudhury, 2019).

Zhu et al. (2019) also studied the functions of the FEX protein in the *Arabidopsis* and tea plants and reported that the tea plant’s CsFEX fluoride export gene participates in F detoxification by heterologous expression. The homologous gene CsFEX, which is largely found in the plasma membrane, can be activated by exogenous fluorine treatment (Zhu et al., 2019). However, it is still unknown how fluoride outflow and buildup work at the molecular level, as well as whether regulatory systems are involved.

According to Do et al. (2021) ABCs (ATP-binding cassettes) transporters control cellular processes by binding ATP and hydrolyzing it to create energy for transport and other physiological and biochemical processes. Eight subfamilies make up the ABC transporter gene family, and the proteins they encode can move a wide range of substrates. ABCs bind ATP and hydrolyze it to provide energy that powers transport and controls additional cellular processes. There are eight subfamilies in the ABC transporter family of genes, and the proteins they code for can transport a wide variety of substrates. As a result, they control almost all physiological and biochemical processes in plants (Do et al., 2021).

A study showed that F increases the concentration of ABC transport proteins in tea trees and by RNA-sequence analysis, the CsABCB9 gene is explored whose expression is boosted by F treatment (Luo et al., 2022).

A study shows that exogenous salicylic acid (SA) has a critical role in mitigating the toxic effects of F in rice seedlings and demonstrates its significant role as a protective molecule against fluoride stress when supplied exogenously (Singh and Roychoudhury, 2021; Lu et al., 2022).

On the other hand, F circulation in the ecosystem of tea plantations is favorable, with more F being added than removed. The main sources of F absorption by tea plant root through active transmembrane transport and anion channels are magnesium chloride (MgCl_2_) extractable F and water extractable F in plantation soil. The majority of F is quickly transferred as F/F-Al complexes to the leaf cell walls and vacuole across the xylem. The results suggest that tea plants detoxify F and aluminum (Al) simultaneously through cell wall accumulation, vacuole compartmentalization, and F-Al complexes, which may be a mechanism of F tolerance that enables tea to withstand higher F concentrations than most plants (Peng et al., 2020; Luo et al., 2022).

According to Luo et al. (2022), tea plants (*Camellia sinensis*) collect a disproportionate quantity of F in their leaves when compared to other plants. However, it is uncertain how these plants tolerate F. A chloroplast F efflux gene (CsABCB9) was identified through transcriptome analysis, cloned from *Camellia sinensis*, and its function in F detoxication was proven in *Escherichia coli* and *Arabidopsis thaliana*. Tea leaves express the CsABCB9 efflux gene after F treatment.

### 3.4. Induction of cyclic electron flow in fluoride tolerance

Singh and Jajoo (2021) investigate the negative effects of F on photosynthesis, specifically in maize plants (*Zea mays* L.) Photosystem I (PSI) and Photosystem II (PSII) serve as the principal locations of energy conversion that convert light energy into chemical energy (Chadha et al., 2021). F has a negative effect on the activity of both photosystems, which serve as internal environmental monitors. In contrast to PSI, Y (I), the quantum yield of PSII, Y (I), was reduced at all NaF concentrations. The activation of cyclic electron flow (CEF) after F treatment was accompanied by the suppression of linear electron flow (LEF). PSI resistance to F poisoning appears to need LEF inhibition and CEF induction (Singh and Jajoo, 2021).

## 4. Fluoride tolerance mechanism of PGPR

F removal is required before using fluoridated water because the content of F exceeds the allowable limits. F tolerance mechanisms in microorganisms vary depending on the specific organism and its adaptation to F-rich environments (Yan et al., 2022; Li et al., 2023). Many organisms have developed unique defense mechanisms for dealing with high F concentrations, for example, the synthesis of proteins capable of eliminating F from cells. However, these F transporters have not been found in all microorganisms and F transporters may vary in their tolerance capabilities between species, individuals, and even tissue types. This shows that the F tolerance capacity of PGPRs is also influenced by other factors.

The following sections describe various F tolerance mechanisms of PGPR including; Efflux pumps, Intracellular sequestration, enzyme modifications, enhanced DNA repair mechanism, detoxification enzymes, ion transporter/ antiporters, fluoride riboswitches, and genetic mutations.

### 4.1. Enzymatic modification for fluoride tolerance

According to the study, maintaining high bacterial biomass is critical in bacterial-based bioremediation for boosting bacterial species capacity and survival in the F environment, this is done by immobilizing bacterial cells (Mukherjee et al., 2018).

The most electronegative element is fluoride, which penetrates bacterial cells through diffusion as hydrogen fluoride, dissociating into H^+^ and F ions (Katiyar et al., 2020). These ions disrupt fluoride-ATPases and glycolysis enzymes. Microorganisms that have F tolerance capacity are thought to have altered enzymes (Waugh, 2019).

Numerous microorganisms have the potential to bioabsorb, biotransform, and bioaccumulate ligands like biosurfactants or siderophores, which impact the availability and solubility of pollutants in bacterial cells (Banat and Thavasi, 2019).

To avoid F toxicity, PGPR must be carried out in ionospheres, mineralization, metal intake, buildup, sorption, reduction, enzymatic oxidation extracellular precipitation, and xenobiotic outflow (Makete et al., 2022).

The bacterium *Bacillus flexus* (PN4) may be a promising strain for defluorinating drinking water, and it may offer a perfect opportunity to develop a novel bioremediation method, according to the findings of a study by Sakthi Thesai et al. (2018). The bacterium might be crucial in the process of removing F from aqueous medium. As a result, there might be a viable remediation method for F-containing water after fluoride accumulation and bacterial eradication.

### 4.2. Intracellular mechanism of fluoride accumulation in bacterial cell

**Figure 4:**
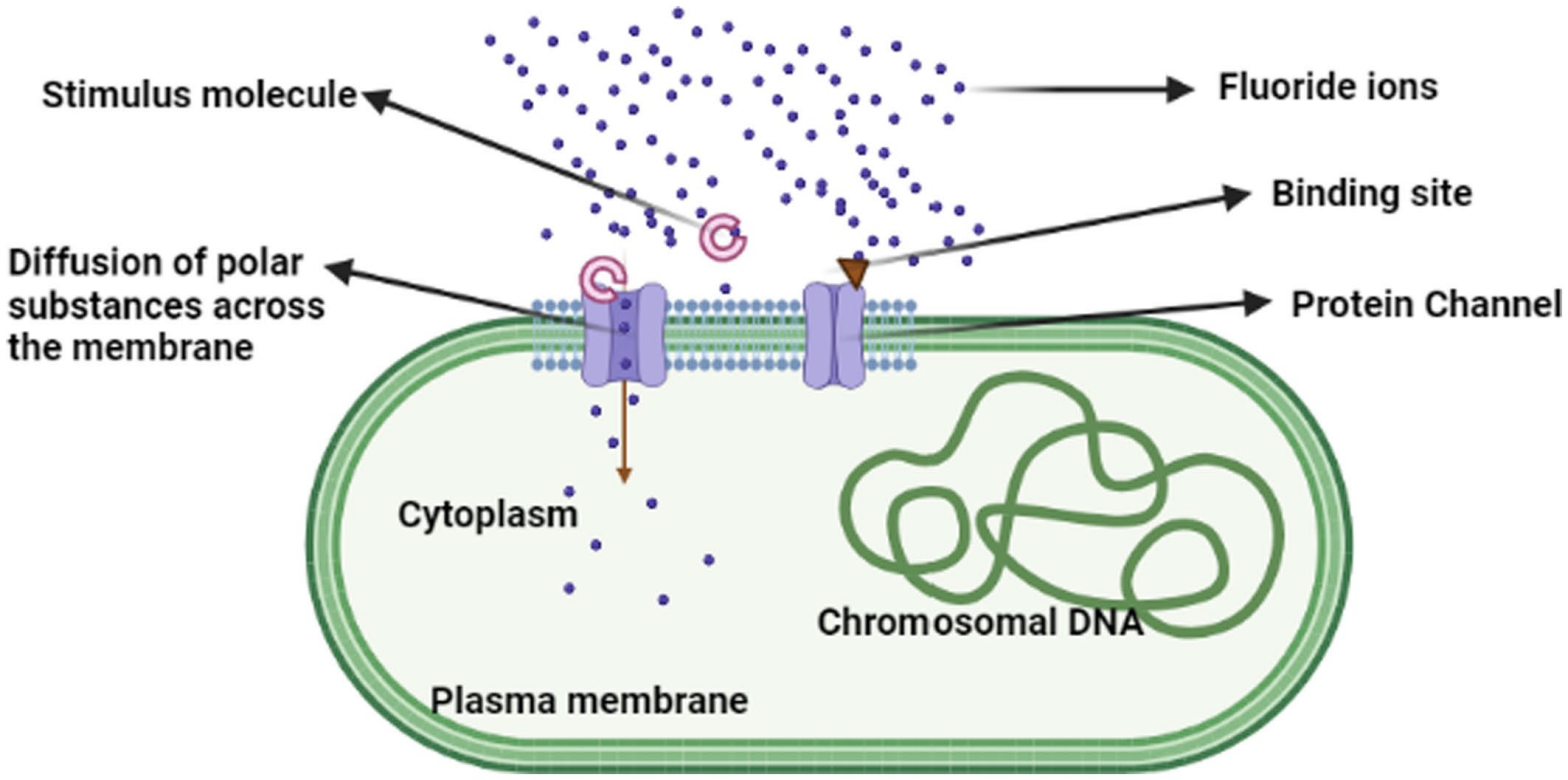
Mechanism of fluoride intracellular accumulation in bacterial cells.

According to Sakthi Thesai et al. (2018) F anion exporters are described by Fluc family F-specific ion channels. F hypersensitivity occurred in the bacteria due to a paucity of potential proteins. Their outcome was that the microbes developed F/H^+^ antiporters of CLC anion transporters, resulting in the emergence of the “Fluc” family of F-specific ion channels. Anionic F was able to enter the bacterial cell through the pore when stimulus molecules attached and stimulated the protein channel (fluc), causing the F ion to export into the bacterial cell (Figure 4). Rhizobacteria can develop fluoride resistance by changing the promoter of fluoride antiporters (Chiariello et al., 2020).

### 4.3. F0F1-ATPase for fluoride tolerance

Li et al. (2022) studied F tolerance in *Streptococcus mutans* induced by high F concentrations via point mutations. They found three F-resistant *S. mutans* strains and termed SRR13846724, SRR13846723, and SRR13846722 for the FR300, FR600, and FR1000. Three types of variant studies were performed on the scaffolds of all strains using whole-genome sequencing: structural variations, insertion–deletion variant (InDel), and calling SNP. There was no evidence of chromosomal rearrangement in the resistant bacteria, despite their great similarity. These findings shed new light on the microbial F tolerance mechanism, F0F1-ATPase is necessary for antagonizing F inhibition and increasing F resistance in *S. mutans* (Li et al., 2022).

Pal et al. (2022) identified soil microorganisms and revealed that these microorganisms are very similar to *Bacillus megaterium* and resistant to solutions containing 35% (w/v) sodium chloride and 1,500 mg/L Fluoride. *Bacillus megaterium* (JF273850), an isolated microbe, may be useful for F protection.

A study suggests that soil bacterium *Pseudomonas putida* KT2440 reacts to F anion provided by Calero et al. (2022). The study uses a broad lens to illustrate how F affects the physiological response of the bacterium and the microorganism.

### 4.4. Genetic modification for fluoride resistance

A study investigates the alteration in genes of *Streptococcus mutans*, which give rise to F-resistant strains, and also explains how genetic mutations cause pleiotropic consequences in the physiology of *S. mutans*. In the F-resistant genome, they discovered six mutations through sequencing analysis (Lee H.-J. et al., 2021; Lee J. et al., 2021). Fluoride stress causes a cumulative effect of genetic changes that may rewire intricate machinery to balance bacterial resistance and biological fitness in the best possible manner (Liao et al., 2018).

In order to determine which genetic mutations primarily cause the resistant phenotype and decreased fitness in *S. mutans*, complementation for each mutation should be carried out.

### 4.5. pH-dependent fluoride tolerance

The pH of the aqueous solution and the functional groups on the microbe cell wall determine the degree of biosorption (El-Naggar et al., 2018). Increasing the pH concentration resulted in decreased fluoride adsorption. This might be because, at higher pH concentrations the amount of OH^−^ is greatest and F, which is extremely electronegative, repels the negatively charged OH^−^ (Nabbou et al., 2019). However, due to the low pH level, there may be more interaction between the negatively charged F and positively charged H^+^, which could result in the formation of hydrofluoric acid (HF) (Abraham and Acree, 2019).

A study suggests that F bioremediation capacity by *Bacillu*s sp. subjected to varied pH. The effectiveness of bioremediation has risen from 40 to 55% at pH 7 to 60–78% at pH 6. When the pH is changed, the absorption efficiency increases from 200 ppm to 286 ppm. Thus, pH has an important function in minimizing environmental pollution and emphasizes the relevance of bacteria in an eco-friendly way (Sahoo et al., 2019).

Shanker et al. (2020) investigated the removal of F by *Acinetobacter* sp. and the findings gave a clear insight into the factors influencing bacterial growth and defluorination. The physical and chemical analysis of *Acinetobacter* sp. showed that after 10 h of incubation at 7.5 pH 57.3% of the F from the synthetic aqueous solutions (Shanker et al., 2020).

According to Mothe et al. (2021) from groundwater samples taken in Narketpally, a heavily fluoridated location in the Nalgonda district, three F-resistant bacteria (MB1, F, and G) with high F resistance (up to 500 mgL^−1^ NaF) were identified. After 8-day incubation, dextrose (10 g) was used as the carbon source per 100 ml of medium, and the concentration of F was 20 mgL^−1^ at 30°C and pH 7 The F and G strains showed the highest F degradation of 57, and 44%, respectively, while the MB1 strain showed maximum F elimination of 68%.

### 4.6. Role of specific single nucleotide polymorphism in fluoride tolerance

Several single nucleotide polymorphism (SNPs) were found in two different F-resistant *Streptococcus mutans* strains (Liao et al., 2018). In two different strains of the F-resistant *S. mutans*, they discovered overlapping chromosomal regions with SNPs by comparing genome sequences. In two intergenic areas (mutp and glpfp) and one pathway (glycolysis), validation discovered changes in gene expression and protein activities. New potential loci for inhibitor resistance are suggested by their findings. More investigation is required into the function and importance of these loci in the regulation of genes in the presence or absence of fluoride. These SNPs have the capacity to modify protein functions and gene expression.

In another study on the establishment of a collection containing mutated genes extracted from *S. mutans*, the identification of fluoride-related transcriptional regulator (FrtR) as a fundamental transcription factor that plays a significant role in the regulation of F. After analyzing the frtR mutant using RNA-sequencing, it was found that the fluoride related permease gene (frtP) was one of the downstream genes that are directly regulated by FrtR (Lu et al., 2020). The mechanism behind intrinsic F tolerance has yet to be fully understood.

### 4.7. Formation of nucleation sites for fluoride tolerance

In a recent study conducted by Su et al. (2020), to determine the effectiveness of the calcium-precipitating strain *Acinetobacter* sp. H12in eliminating F. The study showed that H12 was able to successfully decrease 85.24% of F at a consistent rate of 0.036 mg·L^−1^·h^−1^. The process was thoroughly analyzed using advanced imaging techniques and spectroscopy, which revealed that the bacterium acted as nucleation sites in forming biological crystal seeds. These seeds then proceeded to adsorb F. These findings demonstrate the significant potential of H12 in addressing the issue of F contamination (Su et al., 2020).

A study that delves into the properties and mechanisms of fluoride removal, highlights the use of biomineralizing bacteria, particularly *Acinetobacter* sp. H12, which has proven to be effective in eliminating F (Wu et al., 2021). Additionally, it was found that the carbon-to-nitrogen ratios affected the fluoride removal performance of *Acinetobacter* H12 in a quartz sand-filled biofilm reactor (Ali et al., 2021).

These findings have significant implications in advancing the treatment of F toxicity.

### 4.8. Fluoride-specific crcB (fluoride riboswitches) resistance gene

According to Chellaiah et al., 2021, eight F-resistant bacteria were isolated from the water samples of the South Indian region of Dindigul. This is the first study to identify fluoride-resistant bacteria, specifically in the Nathamtaluk of southern Tamil Nadu. F-resistant strains identified as *Aeromonas caviae* strains 31, 32, and 34, *Enterobacter cloacae* strain 3, *E. hormaechei* strain 14, *Enterobacter* sp. strain 21, *E. hormaechei* strain 22, and *E. coli* strain S2-9. The fluoride resistance of the selected isolates ranged between 200 and 300 mM NaF. F tolerance has also been associated with antibiotic resistance to Chloramphenicol, cefotaxime, doxycycline hydrochloride, fosfomycin, minocycline, nalidixic acid, nitrofurantoin, and trimethoprim. The ‘CrcB’ gene is revealed by them which is linked to bacterial F resistance. Furthermore, these identified microorganisms will be helpful for F bioremediation experiments (Chellaiah et al., 2021).

Twenty-two F-resistant bacteria in LB agar plates were resistant to 200 mM NaF and showed hemolytic activity on blood agar plates. Gene-specific PCR investigation verified the presence of virulence and biofilm-forming genes in *Pseudomonas* sp. Furthermore, a disc diffusion approach revealed that haemolytic *Pseudomonas* was resistant to various medications. By using gene-specific primers, it is concluded that crcB domain is responsible for F-resistant in *Pseudomonas* sp. (Edward Raja et al., 2022).

## 5. Future prospects

Defluoridation procedures could eliminate the difficulties associated with F contamination. Several technologies (reverse osmosis, nanofiltration, precipitation, adsorption, and so on) have been used to defluoridate water. Adsorption techniques are commonly used to remove excessive F from water Tea ash, Banana peel dust, coconut shell dust, Bsh scale dust (hot and semi-arid climate). However, due to significant capital and operating costs, these technologies could not be used in practice. Conventional F cleanup procedures are time-consuming, labor-intensive, and expensive, as a result, they are uneconomical for long-term cultivation. The solution is to reduce the use of modern, affordable, cost-effective, and economical methods to reduce this environmental pollution. In biological processes like bioremediation, the employment of fungi, bacteria, algae, and higher plants has the ability to drastically reduce F pollution, rehabilitate contaminated soil, and restore vegetation. The effectiveness of local natural agents in sustainable agriculture can be increased, improved, and chosen over harmful chemicals. Furthermore, these traditional procedure takes a lot of energy and chemical ingredients. Bioremediation approaches could help to solve these issues. The study of how PGPRs respond to toxicants is a crucial aspect of environmental protection and preservation. In this regard, the emergence of F resistance in PGPRs could potentially serve as a novel defense mechanism. Additionally, plasmid DNA may facilitate F tolerance in PGPRs. Furthermore, genetically engineered PGPRs could prove to be valuable tools for bioremediation efforts. However, it is important to evaluate the potential risks associated with these approaches and take appropriate measures to mitigate them.

## 6. Conclusion

The resilience of microorganisms in harsh environmental conditions is truly remarkable. However, the proliferation of natural F levels in soil can have devastating effects on plant health. Fortunately, recent research has indicated that PGPRs have the ability to endure high concentrations of fluoride, offering a promising solution for bioremediation. They pose protective mechanisms to overcome the high concentration of F along with increasing soil health and plant productivity, therefore they can be a promising solution for F bioremediation. Utilizing genetic engineering to enhance PGPR’s resistance against F and other harmful substances in soil represents a highly viable solution for tackling environmental issues. This approach has tremendous potential for success and warrants further exploration.

## Author contributions

AS: Formal analysis, Investigation, Methodology, Writing – original draft, Writing – review & editing. VY: Conceptualization, Data curation, Investigation, Writing – original draft, Writing – review & editing. HG: Conceptualization, Data curation, Validation, Visualization, Writing – review & editing. LR: Conceptualization, Data curation, Project administration, Software, Writing – review & editing. RC: Conceptualization, Data curation, Formal analysis, Supervision, Writing – review & editing. GS: Conceptualization, Investigation, Validation, Visualization, Writing – review & editing. RV: Formal analysis, Project administration, Resources, Validation, Writing – review & editing. DS: Conceptualization, Funding acquisition, Methodology, Visualization, Writing – review & editing. AP: Conceptualization, Investigation, Visualization, Writing – original draft, Writing – review & editing.
